# Decision of the “Hard Rubber” Contest

**Published:** 1859-12

**Authors:** 


### DECISION OF THE "HARD RUBBER" CONTEST.

By reference to a card in the advertising sheet of the present number of the Register, it will be seen that in the suite of the Hard Rubber Co. *vs.* E. A. L. Roberts, for infringement of Patent Right, has been decided, granting the injunction. An order of investigation has been issued in regard to the amount of work done by the defendant, and an order for the amount of profits upon the same, to be paid by the defendant to the plaintiff.

In regard to the details of the matter, we know nothing more than is stated in the circular. This decision will probably bring the indiscriminate use of this process to a check.

There may be developments that will change the aspect of affairs in future ; of this, however, we know nothing. Those who wish to avoid difficulty, should regard the rights of others. T.

